# A single dose of dapagliflozin, an SGLT-2 inhibitor, induces higher glycosuria in GCK- and HNF1A-MODY than in type 2 diabetes mellitus

**DOI:** 10.1007/s12020-017-1341-2

**Published:** 2017-06-07

**Authors:** J. Hohendorff, M. Szopa, J. Skupien, M. Kapusta, B. Zapala, T. Platek, S. Mrozinska, T. Parpan, W. Glodzik, A. Ludwig-Galezowska, B. Kiec-Wilk, T. Klupa, M. T. Malecki

**Affiliations:** 1 Department of Metabolic Diseases, Jagiellonian University Medical College, Krakow, Poland; 2 Department of Metabolic Diseases, University Hospital, Krakow, Poland; 3 Department of Clinical Biochemistry, Jagiellonian University Medical College, Krakow, Poland; 4 Brothers Hospitallers’ of St. John of God Hospital, Krakow, Poland; 5 Sanatio Medical Center, Krakow, Poland; 6 Center for Medical Genomics OMICRON, Jagiellonian University Medical College, Krakow, Poland

**Keywords:** HNF1A, GCK, MODY, SGLT2, Dapagliflozin

## Abstract

**Aims:**

SGLT2 inhibitors are a new class of oral hypoglycemic agents used in type 2 diabetes (T2DM). Their effectiveness in maturity onset diabetes of the young (MODY) is unknown. We aimed to assess the response to a single dose of 10 mg dapagliflozin in patients with Hepatocyte Nuclear Factor 1 Alpha (HNF1A)-MODY, Glucokinase (GCK)-MODY, and type 2 diabetes.

**Methods:**

We examined 14 HNF1A-MODY, 19 GCK-MODY, and 12 type 2 diabetes patients. All studied individuals received a single morning dose of 10 mg of dapagliflozin added to their current therapy of diabetes. To assess the response to dapagliflozin we analyzed change in urinary glucose to creatinine ratio and serum 1,5-Anhydroglucitol (1,5-AG) level.

**Results:**

There were only four patients with positive urine glucose before dapagliflozin administration (one with HNF1A-MODY, two with GCK-MODY, and one with T2DM), whereas after SGLT-2 inhibitor use, glycosuria occurred in all studied participants. Considerable changes in mean glucose to creatinine ratio after dapagliflozin administration were observed in all three groups (20.51 ± 12.08, 23.19 ± 8.10, and 9.84 ± 6.68 mmol/mmol for HNF1A-MODY, GCK-MODY, and T2DM, respectively, *p* < 0.001 for all comparisons). Post-hoc analysis revealed significant differences in mean glucose to creatinine ratio change between type 2 diabetes and each monogenic diabetes in response to dapagliflozin (*p* = 0.02, *p* = 0.003 for HNF1-A and GCK MODY, respectively), but not between the two MODY forms (*p* = 0.7231). Significant change in serum 1,5-AG was noticed only in T2DM and it was −6.57 ± 7.34 mg/ml (*p* = 0.04).

**Conclusions:**

A single dose of dapagliflozin, an SGLT-2 inhibitor, induces higher glycosuria in GCK- and HNF1A-MODY than in T2DM. Whether flozins are a valid therapeutic option in these forms of MODY requires long-term clinical studies.

## Introduction

Maturity Onset Diabetes of the Young (MODY) is estimated to account for 1–2% of all cases of diabetes, which may correspond to hundreds of thousands of patients in Europe. The most typical clinical features of MODY include early age of diagnosis (usually in the 2nd and 3rd decade of life), positive family history, autosomal dominant mode of inheritance, absence of autoantibodies typical for type 1 diabetes mellitus (T1DM), and lack of obesity [1]. Most MODY cases result from mutations within the hepatocyte nuclear factor 1 alpha (*HNF1A*) and glucokinase (*GCK*) genes [2, 3].

Mutations in the *HNF1A* gene, which encodes a transcription factor that regulates the expression of many other genes, lead to insulin secretion impairment [4]. As a result, patients with HNF1A-MODY require pharmacotherapy and often develop chronic complications [5, 6]. These patients typically respond well to treatment with sulfonylurea, which increases insulin release from beta cells and is currently considered the treatment of choice for patients with HNF1A-MODY [7, 8]. As the nature of the beta-cell defect in HNF1A-MODY is progressive, this treatment frequently requires further intensification with additional hypoglycemic agents. The extra-pancreatic features of HNF1A-MODY include glycosuria due to a low renal threshold for glucose [9], which has been linked to decreased sodium/glucose co-transporter 2 (SGLT2) expression in tubular cells [10].

Meanwhile, in GCK-MODY glucose homeostasis is less affected than in HNF1A-MODY. GCK-MODY subjects are characterized by mild fasting hyperglycemia. It is generally accepted that most diabetic *GCK* mutation carriers do not need hypoglycemic treatment [11]. They usually do not develop advanced chronic microvascular complications of diabetes, although, the prevalence of background retinopathy is rather high, reaching 30% according to British data [12]. Moreover, there is also some evidence on abnormalities in surrogate cardiovascular outcomes in *GCK* mutation carriers [13]. Therefore, clinicians would probably consider initiation of treatment in some GCK-MODY patients. Regrettably, pharmacological treatment including either insulin, or oral hypoglycemic agents, such as metformin or SU, turned out to be ineffective in this form of MODY, probably due to the fact that the switch-on of the counter-regulatory response occurs at higher glucose level than in healthy subjects [14, 15]. So, there is still a need to test new available treatments in both of the two most frequent subtypes of MODY [1]. This is particularly important because thanks to the widening use of next-generation sequencing a growing number of patients with a genetic diagnosis of MODY will be identified [16, 17].

Recently a new group of oral hypoglycemic agents—SGLT2 inhibitors—were introduced into the market and made available for patients with diabetes. These inhibitors block the low-affinity, high capacity glucose transporter located in the proximal tubule in the kidneys that is responsible for 90% of glucose reabsorption. Therefore, SGLT2 inhibitors can reduce plasma glucose levels by producing glycosuria [18]. A single dose of the SGLT2 inhibitor dapagliflozin was shown to be enough to produce glycosuria and the effect lasted up to 24 h [19, 20]. As mentioned above, the expression of *SGLT2* and the function of the SGLT2 protein are reduced among HNF1A-MODY individuals [10]. Therefore, it can hypothesized that response to SGLT2 inhibitors, such as dapagliflozin, may be compromised among HNF1A-MODY individuals, thus influencing their efficacy in these patients.

## Aim of the study

We aimed to assess the response to a single dose of 10 mg dapagliflozin in patients with HNF1A-MODY and to compare it with T2DM and GCK-MODY individuals by measuring changes in urinary glucose to creatinine ratio (GCR) and in serum 1,5-anhydroglucitol (1,5-AG) level, which indirectly reflects episodes of glycosuria. 1,5-AG is a short-term marker of glycemic control (mainly postprandial) that corresponds to the preceding 1–2 weeks. In a state of glycosuria, renal reabsorption of 1,5-AG is decreased, as this particle competes with glucose for reabsorption in the proximal renal tubule, which results in its lower serum level [21–24]. Additionally. we assess GCR change as a diagnostic tool to distinguish between T2DM and MODY.

## Materials and methods

### Study population

The studied group included 14 HNF1A-MODY patients, 19 GCK-MODY individuals, and 12 T2DM subjects. All MODY patients had a heterozygous loss-of-function mutation either in the *HNF1A* or *GCK* gene identified by direct DNA sequencing. HNF1A-MODY patients were members of 13 families, whereas GCK-MODY individuals were members of 17 families. T2DM individuals were ascertained as described earlier [25].

We included patients with at least 2 years duration of diabetes and without diabetic kidney disease defined as Chronic Kidney Disease Epidemiology Collaboration (CKD-EPI) estimated glomerular filtration rate (eGFR) <60 ml/min/1.73 m^2^ diagnosed prior to inclusion in the study. We excluded patients with a hemoglobin A1c (HbA1c) >9.0% (75 mmol/mol), those who were on insulin or steroid therapy, or taking drugs affecting renal function (e.g., loop diuretics). Pregnancy was also an exclusion criterion. We gathered clinical data on the study individuals from their medical records. Additionally, we collected the information from the standard questionnaire and performed a basic physical examination. All studied individuals received a single morning dose of 10 mg of dapagliflozin. The patients’ current therapy, including use of other hypoglycemic agents, was not changed. To assess the glycosuric response to dapagliflozin the following analyses were performed in all subjects: (1) three measurements of urinary glucose and creatinine concentrations: one in the morning on the dapagliflozin administration day (*Day 0*), one 8–12 h after administration of the drug, and one in the morning on *Day +1*; (2) two measurements of serum 1,5-AG level: one in the morning on *Day 0* and one in the morning on *Day +1*. Additionally, we performed two measurements of fasting plasma glucose (FPG): one in the morning on *Day 0*, and one in the morning on *Day +1*, as well as two daily glucose profiles performed with glucose meters by participants (fasting, before and 2 h after main meals): one on the day before drug administration (*Day −1*) and one on the dapagliflozin administration day. Urine glucose concentration below 1.0 mmol/l was considered negative. Urinary GCR was calculated for all three urine measurements. 1,5-AG was measured in fasting conditions using a competitive inhibition enzyme immunoassay (ELISA; Cloud Clone Corp., Houston, TX 77,082, USA). The assay has a sensitivity of 0.7 µg/ml, the intra- and inter-assay coefficients of variation of the ELISA were <10%. The absorbance was finally measured in a microplate reader (BioTek Instruments, Winooski, VT, USA). The study was approved by the local Bioethics Committee. All participants gave written informed consent.

### Statistical analysis

Statistical analysis was performed using Statistica Software v. 12.0 (StatSoft, Tulsa, OK, USA) and MedCalc Statistical Software v 16.8.4 (MedCalc Software bvba, Ostund, Belgium). All performed tests were two-tailed and a *p*-value <0.05 was considered significant. The Kolmogorov-–Smirnov test was used to determine the normal distribution of variables. *p*-values were calculated with the *t*-test and the one-way ANOVA or nonparametric Kruskall–Wallis test, followed by post hoc-tests. Multivariate linear regression analysis (backward selection) was used to assess differences in mean GCR and 1,5-AG change between studied groups after adjustment for gender, age, diabetes duration, body mass index (BMI), eGFR, FPG, and HbA1c. Diagnostic performance (i.e., the ability of GCR after dapagliflozin administration to identify MODY and T2DM) was assessed using the receiver operating characteristics (ROC) curve. The standard error (SE) of the area under the ROC curve (AUC) and 95% confidence intervals (95% CI) were calculated using DeLong’s method [26].

## Results

The characteristics of the study participants are presented in Table 1. There were differences between the study groups for age, BMI and HbA1c. The patients with T2DM were older, more obese, and were characterized by worse glycemic control than HNF1A-MODY and GCK-MODY subjects what is in line with the way how groups were defined. In post-hoc analyses, we found no differences between HNF1A-MODY and GCK-MODY subjects according to age, BMI and HbA1c. There were no differences between study groups in terms of sex distribution and kidney function. During the study examination we diagnosed one patient with chronic kidney disease (eGFR 47 ml/min/1.73 m^2^) and his results were included in the analyses. Results presented by that patient were not detected as outliers. All study participants were free from proliferative diabetic retinopathy. Almost all *GCK* gene mutation carriers were on dietary therapy only; only one GCK-MODY patient (a 67-year old female) was on metformin. Among the 14 HNF1A-MODY individuals, 12 were on SU, one patient (a 30-year female with polycystic ovary syndrome) was on metformin, and one was on dietary therapy only. Slightly more than half of patients with T2DM were on combined therapy with metformin and SU or DPP4 inhibitors; other T2DM patients were on metformin monotherapy.

**Table 1:** Clinical characteristic and biochemical measurements of the study group

Characteristic	HNF1A-MODY	GCK-MODY	T2DM	*p*-value
*N*	14	19	12	NA
Male/female (*n*)	7/7	7/12	8/4	0.17^a^
Age at examination (years)	34.1 ± 11.0	40.3 ± 10.8	61.8 ± 5.6	0.0000^b^
Time from diabetes diagnosis (years)	11.4 ± 6.8	8.7 ± 7.1	5.8 ± 4.2	0.10^b^
BMI (kg/m^2^)	24.4 ± 4.9	23.4 ± 2.8	31.3 ± 5.3	0.0000^b^
eGFR CKD-EPI (ml/min/1.73 m^2^)	98.1 ± 15.1	99.9 ± 15.6	87.7 ± 15.9	0.10^b^
HbA1c (%, mmol/mol)	6.0 ± 0.7, 42.0 ± 5.3	6.4 ± 0.4, 46.0 ± 2.0	6.9 ± 0.9, 52.0 ± 7.5	0.03^c^
hsCRP (mg/l)	0.52 ± 0.30	1.12 ± 1.57	1.60 ± 1.15	0.09^b^
GCR (*Day 0*)—patients with positive urinary glucose only (mmol/mmol, *n*)	0.15, 1	0.17 ± 0.18, 2	2.01, 1	NA
GCR change (8–12 h after dapagliflozin administration) (mmol/mmol)	35.64 ± 14.10	38.72 ± 9.65	19.37 ± 18.76	0.001^b^
GCR change (*Day +1*—*Day 0*) (mmol/mmol)	20.51 ± 12.08	23.19 ± 8.10	9.84 ± 6.68	0.001^b^
1,5-AG (Day 0) (mg/ml)	8.75 ± 8.43	19.61 ± 9.22	26.72 ± 9.03	0.0000^b^
1,5-AG change (Day +1 −Day 0) (mg/ml)	−0.09 ± 4.21	−0.86 ± 4.16	−6.57 ± 7.34	0.02^b^
FPG (Day 0) (mmol/l)	5.60 ± 1.02	6.82 ± 0.71	7.73 ± 2.25	0.005^c^
FPG change (Day +1—Day 0) (mmol/l)	−0.14 ± 0.90	−0.50 ± 0.72	−0.23 ± 1.20	0.51^b^
Average daily glucose in SMBG (Day −1) (mmol/l)	6.02 ± 1.03	6.99 ± 0.50	7.60 ± 1.77	0.008^c^
Average daily glucose in SMBG (Day 0) (mmol/l)	5.81 ± 0.87	6.90 ± 0.59	7.32 ± 1.58	0.002^c^
Average daily glucose change in SMBG (Day 0—Day −1) (mmol/l)	−0.21 ± 0.61	−0.09 ± 0.51	−0.27 ± 1.27	0.84^c^

Data are presented as mean ± standard deviation

*1,5-AG* 1,5-Anhydroglucitol, *FPG* fasting plasma glucose, *GCR* glucose to creatinine ratio, *hsCRP* high sensitive C-reactive protein, *NA* not applicable, *n* number of cases, *SMBG* self-monitoring of blood glucose

^a^ χ^2^ test

^b^ One-way analysis of variance (ANOVA)

^c^ Kruskal–Wallis test

There were only four patients with positive urine glucose before dapagliflozin administration—one patient with HNF1A-MODY, two subjects with GCK-MODY, and one individual with T2DM. After 8–12 h of dapagliflozin administration glycosuria occurred in all studied patients and the effect persisted for 24 h in every participant. Significant differences in urinary GCR after 24 h of dapagliflozin administration were observed in the studied groups as this rose by 20.51 ± 12.08, 23.19 ± 8.10, and 9.84 ± 6.68 mmol/mmol for HNF1A-MODY (*p* = 0.0000), GCK-MODY (*p* = 0.0000), and T2DM (*p* = 0.0003), respectively. These mean changes in urinary GCR were different between the groups (*p* = 0.001). Post-hoc analysis revealed significant differences in mean GCR rise between the T2DM and MODY forms of diabetes (*p* = 0.02 and *p* = 0.003 for HNF1-MODY and GCK-MODY, respectively); however, no difference between the HNF1A-MODY and GCK-MODY groups were observed (*p* = 0.72). Nevertheless, in multivariate linear regression (R2 = 27%), the only significant variable remained BMI (*p* = 0.0001), but not type of diabetes. Similar results in GCR change in the evening on *Day 0* (after 8–12 h of dapagliflozin administration) were observed.

As expected, mean 1,5-AG level on *Day 0* was the lowest in HNF1A-MODY. The significant change in mean serum 1,5-AG was noticed only in T2DM, for which it was −6.57 ± 7.34 mg/ml (*p* = 0.04), but not in HNF1A-MODY (−0.10 ± 4.21 mg/ml, *p* = 0.94) or GCK-MODY (−0.86 ± 4.16 mg/ml, *p* = 0.38). In the one-way ANOVA, a considerable difference in mean 1,5-AG change among the studied groups was found (*p* = 0.02). Post-hoc analyses revealed significant discrepancy in 1,5-AG change between T2DM and HNF1A-MODY (*p* = 0.04) and a borderline difference between T2DM and GCK-MODY (*p* = 0.07). In the multivariate linear regression (R2 = 27%), the only significant variable was initial serum 1,5-AG level (*p* = 0.0009).

Mean initial FPG was significantly lower in the HNF1-MODY group than in the GCK-MODY and T2DM groups. There were no significant differences in FPG between the GCK-MODY and T2DM groups. Subgroup analyses revealed significant mean FPG reduction in GCK-MODY (0.50 ± 0.72 mmol/l, *p* = 0.007), but not in HNF1A-MODY (0.14 ± 0.90 mmol/l, *p* = 0.56) and T2DM (0.23 ± 1.20 mmol/l, *p* = 0.52). ANOVA analysis revealed no differences in the degree of FPG reduction at this sample size.

Mean initial average daily glucose level based on self-monitoring of blood glucose (SMBG) was the lowest in the HNF1A-MODY (*p* = 0.01). No significant reduction in mean average daily glucose was found in the studied groups (−0.21 ± 0.61 mmol/l, *p* = 0.2154; −0.09 ± 0.51 mmol/l, *p* = 0.4686; −0.27 ± 1.27 mmol/l, *p* = 0.4710 for HNF1A-MODY, GCK-MODY and T2DM, respectively).

**Fig. 1:**
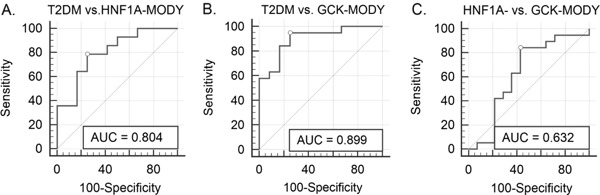
Receiver operating characteristics (ROC) curves showing the discriminative accuracy of glucose to creatine ratio (GCR) change after dapagliflozin administration to distinguish between the diabetes subgroups: **a**.T2DM and HNF1A-MODY; **b** T2DM and GCK-MODY; and **c** HNF1A-MODY and GCK-MODY

Additionally, we assessed change in GCR (after 24 h of dapagliflozin administration) as a biomarker in differential diagnosis for subtypes of diabetes. The ROC curves showing the ability of GCR change to differentiate between the three subtypes of diabetes are presented in Fig. 1. The ROC curve analysis performed for T2DM and HNF1A-MODY revealed an AUC of 0.80 (95% CI 0.60–0.93) with sensitivity 78.6% (95% CI 49.2–95.3) and specificity 75.0% (95% CI 42.8–94.5). The results for T2DM and GCK-MODY were: 0.90 (0.74–0.98), 94.7% (74.0–99.9) and 75.0% (42.8–94.5), respectively. For GCK-MODY and HNF1A-MODY, AUC was 0.63 (95% CI 0.45–0.79), with a sensitivity of 84.2% (95% CI 60.4–96.6%) and specificity of 57.1% (95% CI 28.9–82.3%).

## Discussion

In this small-scale study, we assessed the response to a single morning dose of 10 mg of dapagliflozin in HNF1A-MODY patients and compared to T2DM and GCK-MODY subjects. SGLT2 inhibition was previously shown to be clinically effective, safe and reduce cardiovascular outcomes and mortality as well as death from any cause in T2DM patients. Desirable effects of SGLT2 inhibitors include no risk of hypoglycemia, body weight loss and a small reduction of systolic blood pressure. The most common side effects are genitourinary infections [27–30]. The SGLT2 inhibitor dapagliflozin also induces glycosuria in healthy volunteers [19, 31]. While there was one case report on a patient undergoing combined therapy with SU and dapagliflozin in ABCC8-MODY [32], to the best of our knowledge, no published studies have investigated the effect of SGLT2 inhibitors in patients with GCK-MODY or HNF1A-MODY. Here, in this short, 24 h clinical experiment, we show for the first time that dapagliflozin induces glycosuria in both HNF1A-MODY and GCK-MODY patients and reduces FPG in GCK-MODY.

Our finding concerning dapagliflozin-induced glycosuria in *HNF1A* mutation carriers is especially interesting. Glycosuria in HNF1A-MODY results from reduced expression of the *SGLT2*, which is under the transcriptional control of HNF1A. In HNF1A-deficient animals, there is 80–90% reduction in *SGLT2* expression, which correlates with a reduction in SGLT2 activity [10]. Furthermore, glycosuria that occurs in HNF1A-deficient animals cannot be compensated by the activity of other glucose transporters such as SGLT1 (sodium/glucose co-transporter 1) and SGLT3 (sodium/glucose co-transporter 3) [10]. SGLT2 is responsible for 90% of glucose reabsorption. However, in humans, SGLT2 inhibitors block only 30–50% of the glucose load, and a number of explanations have been proposed to explain this phenomenon [33, 34]. One potential cause of this effect is that SGLT1 works at submaximal capacity under physiological conditions, with the residual capacity of SGLT1 revealed upon SGLT2 inhibition [35]. However, the precise mechanisms of incomplete inhibition of glucose load with SGLT2 inhibitors and the interplay between SGLT1 and SGLT2 transporters are not fully understood. In our study, we observed no difference between *HNF1A* and *GCK* mutation carriers in the effects of dapagliflozin administration. This may mean that the degree of SGLT2 inhibition with dapagliflozin does not depend on baseline activity of the SGLT2 protein, which is lower in HNF1A-MODY patients. Surprisingly, the GCR change was greater in both the HNF1A-MODY and GCK-MODY group than in the T2DM group in our study. However, one must take into consideration the significant clinical differences observed among T2DM patients and those with MODY in our study. Indeed, when we subsequently performed a multivariate linear regression analysis, BMI was the only significant predictive variable for GCR. Due to obvious differences in the way the study groups were defined, we were unable to match our T2DM and MODY patients according to age and BMI.

Serum 1,5-AG level is a short-term marker of glycemic control that competes with glucose for reabsorption [36]. It was shown that 1,5-AG level is reduced in HNF1A-MODY; however, its discriminative accuracy is not high enough for it to be widely used in clinical practice [24, 37]. In our study mean serum 1,5-AG level change after dapagliflozin administration was statistically significant only in T2DM; this finding is in contrast with change in glycosuria, which was lowest in that group. Multivariate linear regression showed that 1,5-AG level change depends on initial 1,5-AG level—which was higher in T2DM than in both MODY forms. A lower change in MODY could also results from more stable glycemia over a period of a day. Moreover, while 1,5-AG level is an independent marker of glycemic control and glycosuria over 7–10 days, in our 24-h study the full impact of dapagliflozin on the level of this marker could not be seen due to the short-time nature of the experiment.

A proper diagnosis of MODY is crucial in order to conduct adequate treatment. While genetic testing becomes more and more available, there is still a need for cheap, easy biochemical markers. Some of them were reported in earlier research (apo-M, hs-CRP, 1,5-anhydroglucitol, ghrelin) [24, 38–40], nevertheless, none of them have been introduced to the clinical practice. Drug response-based diagnostic tests are quite common in medicine, especially in endocrinology, for example, dexamethasone suppression tests, the clonidine test, or the metoclopramide test. While our drug-based test results are promising, one should be cautious with expectations of using the test in routine practice.

Furthermore, we did not evaluate the long-term clinical efficacy of dapagliflozin in MODY patients in the present study. However, we did prove that dapagliflozin works in MODY patients as well as in those with T2DM. Therefore, SGLT2 inhibitors should be considered as an additional therapeutic option in MODY patients in future. A safety aspect that will have to be examined is a possible rise in ketogenesis in these individuals, a phenomenon that was earlier reported in T2DM and T1DM [41]. This seems to be particularly important in HNFA1-MODY, as a few cases of diabetic ketoacidosis were reported in this monogenic diabetes [42, 43].

Interestingly, we observed a significant drop in FPG in GCK-MODY patients after one tablet of dapagliflozin. It is a well known phenomenon that all pharmacological treatment used so far in this form MODY was ineffective due to counterregulatory mechanisms [11, 14, 15]. Most GCK-MODY patients do not need any hypoglycemic treatment. However, clinicians would probably consider treatment initiation in some GCK-MODY individuals, especially in patients with relatively common nonproliferative retinopathy, those with HbA1c >7.0% (53.0 mmol/mol) and/or “overlapping” type 2 diabetes. Thus, identification of an effective and safe drug for these subjects would be of clinical value. It can be hypothesized that SGLT-2 inhibitors could effectively lower blood glucose level in these patients due to their specific mode of action.

## Conclusion

To summarize, we have shown that a single dose of dapagliflozin, an SGLT-2 inhibitor, induces higher glycosuria in HNF1A- and GCK-MODY than in T2DM. There was no difference in response to dapagliflozin between *HNF1A* and *GCK* mutation carriers, despite a previously reported reduction in SGLT2 expression among HNF1A-MODY individuals. Whether flozins, such as dapagliflozin, are a valid therapeutic option as an adjunctive therapy in these MODY patients requires long-term clinical studies.

## Abbreviations

1,5-AG: 1,5-Anhydroglucitol
apo-M: Apolipoprotein M
BMI: Body mass index
CKD-EPI: Chronic kidney disease epidemiology collaboration
eGFR: Estimated glomerular filtration rate
FPG: Fasting plasma glucose
GCK: Glucokinase
GCR: Urinary glucose to creatinine ratio
HbA1c: Hemoglobin A1c
HNF1A: Hepatocyte nuclear factor 1 alpha
hs-CRP: High-sensitivity C-reactive protein
MODY: Maturity onset diabetes of the young
SGLT2: Sodium/Glucose co-transporter 2
SU: Sulfonylurea
T1DM: Type 1 diabetes mellitus
T2DM: Type 2 diabetes mellitus
